# Henry Head (1861–1940) und seine Bedeutung für die Neurologie

**DOI:** 10.1007/s00115-023-01556-7

**Published:** 2023-10-12

**Authors:** Andreas Rheinländer, Markus Weih

**Affiliations:** 1 Praxis Dr. Gehlmann, Berlin, Deutschland; 2 Friedrich-Alexander-Universität Erlangen-Nürnberg, Nürnberg, Deutschland; 3 MVZ Medic Center Nürnberg, Schweinauer Hauptstraße 43, 90441 Nürnberg, Deutschland

**Keywords:** Schmerzübertragung, Head-Zonen, Dermatome, Psychosomatik, Aphasie, Referred pain, Head zones, Dermatome mapping, Psychosomatic medicine, Aphasie

## Abstract

Henry Head ist vielen Neurolog*innen heute vor allem als Namensgeber der Head-Zonen bekannt. Das Konzept, das heute mehr in der Physiologie als in der inneren Medizin bzw. Psychosomatik gelehrt wird, wurde von Head aber höchstwahrscheinlich anders verstanden. Zudem kann als gesichert gelten, dass die Zeichnungen der Head-Zonen nicht von ihm stammen. Aus neurologischer Sicht ist Head heute aus zweierlei Hinsicht wichtig: Sein Selbstexperiment zur Läsion und Regeneration eines peripheren Nervs von 1909 war heroisch. Es hat Generationen von Neurolog*innen geholfen, die Pathophysiologie besser zu verstehen und damit die Prognose peripherer Nervenverletzungen besser einschätzen zu können. Der zweite Beitrag Heads betrifft die radikuläre Organisation auf Rückenmarksebene. Die spezielle Pathophysiologie der Herpes-zoster-Radikulitis erlaubte es ihm, auf der Basis von Vorarbeiten, um 1900 das Konzept des Dermatoms zu entwickeln. Henry Heads Beitrag bestand darin, die Literatur zusammenzutragen und durch eigene Fälle zu ergänzen. Da er zu dieser Zeit der bedeutendste Neurologe zumindest in der englischsprechenden Welt und mit der deutschen Neurologie gut vernetzt war, verbreiteten sich seine Dermatomkarten vermutlich besser als bis dahin bekannte Abbildungen. Weniger erfolgreich war Head aus heutiger Sicht in der Neuropsychologie bzw. mit holistischen Konzepten zu höheren kognitiven Funktionen. Sein Spätwerk zur Aphasie gilt heute als widerlegt. Heads Kritik an der strikten Lokalisation entsprach dabei dem Zeitgeist des beginnenden 20. Jahrhunderts. Dass die klassischen Beispiele Broca- und Wernicke-Aphasie tatsächlich nicht so leicht zu trennen sind, beruhte jedoch mehr auf der Leistung von Generationen von Neurolog*innen und Neuropsycholog*innen folgender Generationen sowie technischen Fortschritten.

## Abstract (en)

Nowadays, Henry Head is best known for his Head zones. The concept was understood very differently by Head in comparison to what current medical books falsely describe them to be. In reality, there is no direct relationship between one particular skin zone and one single organ. It is certain that the drawings considered depictions of the Head zones in today’s medical textbooks were actually not created by Head. From a neurological point of view, Head is important for two reasons: his self-experiment in 1909 to damage one of his own peripheral nerves followed by regeneration was heroic. It has helped generations of neurologists to have a better understanding of the pathophysiology of peripheral nerve damage and thus make a better assessment of the prognosis of such injuries. Head’s second contribution pertains to the radicular organization at the level of the spinal cord. The pathophysiology of herpes zoster radiculitis enabled him to develop the concept of the dermatomes on the basis of preliminary work around 1900. Henry Head’s contribution was the systematic compilation of the existing publications of the time and amendment of his own cases. As he was the most important neurologist at that time, at least in the English speaking world, and was well connected with people in the German neurology community, it was probably easy for him to make his dermatome maps well known. In retrospect, Head was less successful in neuropsychology with holistic concepts for higher cognitive functions which were in vogue during his lifetime. His late work on aphasia is now considered refuted. Head’s criticism of the strict localization was well in syncronization with the zeitgeist of the early twentieth century. Establishing the fact that Broca’s aphasia and Wernicke’s aphasia are not easily diagnostically distinguishable from each other was more an achievement of subsequent generations of neurologists and neuropsychologists as well as technical advances.

Vor etwa 100 Jahren war Henry Head einer der einflussreichsten Neurolog*innen. Seine Arbeit ist jedoch nicht lediglich eine Fußnote in Geschichts- und Lehrbüchern, sondern prägt bis heute zum Teil die klinische Neurologie, teils die Psychosomatik und die innere Medizin. Da Head und seine Arbeiten heute vielen nicht mehr vertraut sind, sollen seine größten Errungenschaften in der Neurologie, aber auch seine Misserfolge aus heutiger Sicht genauer dargestellt werden. Dieser Aufsatz beleuchtet seine Arbeit, sein Wirken und seinen Einfluss auf die klinische Neurologie der Gegenwart.

## Biografie

**Figure 1:**
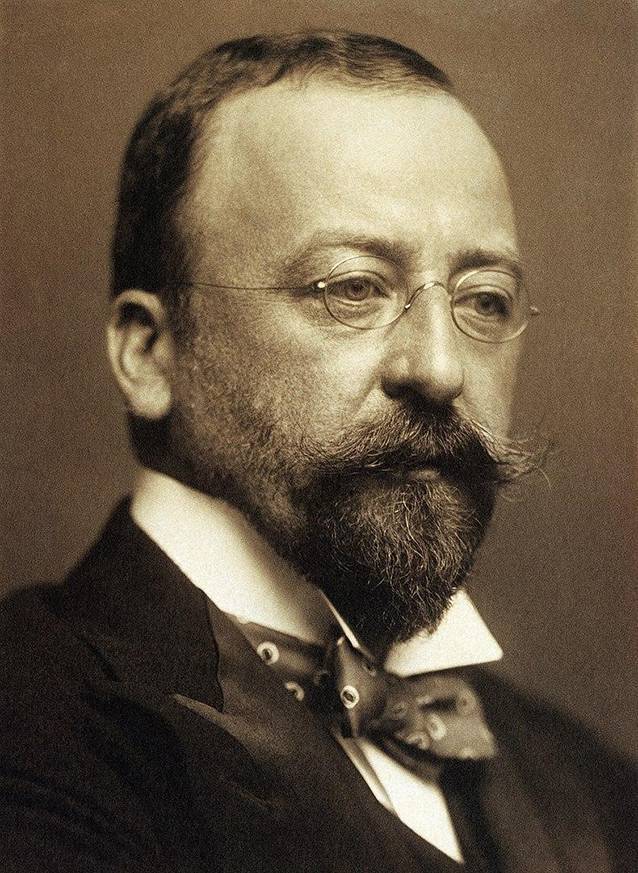

Henry Head (Abb. 1) wurde 1861 als ältester Sohn einer Quäkerfamilie in England geboren.

Er erhielt eine naturwissenschaftliche Ausbildung u. a. in Biologie, Histologie und Physiologie. Da in der naturwissenschaftlichen Medizin und Forschung Deutschland damals führend war, führten ihn Studienaufenthalte nach Berlin bzw. Halle zu Julius Bernstein (1839–1917), Wilhelm His (1831–1904) nach Leipzig und für zwei Jahre nach Prag zu Ewald Hering (1834–1918).

Im Rahmen seiner medizinischen Ausbildung wurde seine Neugier für Infektionskrankheiten wie Scharlach und Gürtelrose geweckt, er beschäftigte sich aber auch mit der Atmung, kardiopulmonalen Erkrankungen und dem Farbensehen. Zurück in England ging er 1886 bis 1890 an das Londoner University College Hospital und praktizierte am National Hospital, Queen Square.

Nervenärztlich tätig war er im Rainhill Asylum, Lancashire. Beteiligt an seiner späteren Ausbildung waren Michael Foster (1836–1907), John Langley (1852–1925), Walter Gaskell (1847–1914), Charles Sherrington (1857–1952) am Trinity College, Cambridge und vor allem natürlich John Hughlings Jackson (1835–1911), dessen Position er 1894 einnahm [1]. Head wurde 1919 vorzeitig emeritiert, als sich Symptome einer Parkinson-Erkrankung zeigten. Er starb 1940.

## Head und die Schmerzübertragung

Dem Medizinstudierenden der heutigen Zeit begegnen die Head-Zonen irgendwann im Verlauf des Studiums – in der Regel im 1. oder 2. Semester in der Anatomie oder Physiologie. Dabei sind zwei Dinge auffällig. Zum einen wird den Zonen eine diagnostische Bedeutung zugeschrieben. Welche diese genau ist und vor allem wie die Anwendung der Erkenntnisse in der Praxis auszusehen hat, ist in der Regel nicht weiter beschrieben. Inzwischen ist klar, dass die heutzutage in Lehrbüchern vorzufindende Darstellung der „Head-Zonen“ nicht viel mit der Arbeit von Henry Head zu tun hat. Die Abbildung stammt vielmehr von einem deutschen Chirurgieassistenten namens Otto Kleinschmidt [2]. Dieser wiederum verwendete eine Zeichnung von James Mackenzie, dessen Name in aktuellen Lehrbüchern in der Regel nicht genannt wird. Mackenzie war ein schottischer Kardiologe, der zu dieser Zeit ebenfalls in London praktizierte. Auch er beschäftigte sich mit verschiedenen Aspekten des Schmerzes, unter anderem der Schmerzübertragung [3]. Head und Mackenzie forschten unabhängig voneinander, lernten sich allerdings zu einem späteren Zeitpunkt kennen [4].

Im Gegensatz zu Head befragte Mackenzie Patient*innen mit Erkrankungen der inneren Organe nach ihrem Spontanschmerz und untersuchte sie sowohl auf oberflächlichen Hautschmerz als auch auf Palpationsschmerz. Auch er fertigte Zeichnungen an, die den Zusammenhang zwischen erkranktem innerem Organ und beschriebenen Schmerzzonen darstellen.

Die Organerkrankungen beschrieb er anhand von Fallbeispielen, zudem fertigte er für einige (aber nicht alle) Abbildungen an, in denen die befundete Hyperalgesie flächig eingezeichnet war. 1914 erschien die erste deutsche Auflage eines populären englischen Chirurgiebuchs [5]. Sie wurde sprachlich angepasst und neu illustriert. Eine Abbildung ist beschriftet als „Ausbreitung der Interkostalnerven und Beziehungen derselben zu den inneren Organen“. Sie wurde von Kleinschmidt (unter der Herausgeberschaft von Erwin Payr [2]) angefertigt. Die Verwertungsrechte für das Bild liegen heute bei seinen Erben [4].

Die Abbildung gleicht den Abbildungen, die heute als „Head-Zonen“ zu finden sind. Die heute als Head-Zonen bezeichneten Areale gehen also auf Otto Kleinschmidt bzw. James Mackenzie zurück. Die von Mackenzie in seinen Darstellungen eingezeichneten Flächen entsprechen grob dem, was heutzutage als Head-Zonen bezeichnet wird. Das Problem, dass die Head-Zonen über die Zeit in verschiedenen Lehrbüchern in verschiedener Form wiedergegeben wurden, wurde in neuerer Zeit mit Methoden der evidenzbasierten Medizin auf eine einheitliche Körpervorlage standardisiert [6]. Das verdeutlicht, dass die klinisch-diagnostischen Konsequenzen, die sich daraus ergeben, von aktueller und praktischer Bedeutung sind. Das Phänomen des übertragenen Schmerzes, der im deutschsprachigen Raum mit dem Begriff der „Head-Zonen“ in Verbindung gebracht wird, wurde erstmals im 19. Jahrhundert verwendet. Die Literatur dieser Zeit lässt sich in zwei Gruppen einteilen: einzelne Fallberichte und große Übersichtsarbeiten. Zu letzteren zählen auch die Werke von Henry Head und James Mackenzie.

Gegen Ende des 19. Jahrhunderts arbeitete Head als Wissenschaftler in verschiedenen Ländern. Während dieser Zeit verfasste er die Abhandlung „On disturbances of sensation with special reference to the pain of visceral disease“ [7]. Darin beschäftigte er sich mit hyperalgetischen Hautzonen bei verschiedenen Erkrankungen der inneren Organe. In dieser Zeit untersuchte Head Patient*innen, die an einer Erkrankung der inneren Organe litten, indem er mit einem Stecknadelkopf über die Haut strich. Anschließend ließ er sich berichten, wo dieser Reiz als schmerzhaft empfunden wurde. Mithilfe dieser Befunde erstellte er topographische Karten, worin bestimmten Organerkrankungen charakteristische Zonen kutaner Hyperalgesie zugeordnet sind – die eigentlichen Head-Zonen. Innerhalb der Hyperalgesiezonen fand er kleine Areale, deren Schmerzhaftigkeit von Patient*innen am stärksten empfunden wurde. Diese Bereiche werden als Maximalpunkte bezeichnet.

Aus historischer Sicht ist heute also einigermaßen klar, wann und wer die später breit reproduzierten Abbildungen erstellt hat. Unklar bleibt, ab welchem Zeitpunkt begonnen wurde, die Abbildung Kleinschmidts als Darstellung der Head-Zonen zu bezeichnen.

Die fälschliche Bezeichnung der Kleinschmidt-Abbildung als „Head-Zonen“ führte unserer Ansicht aber dazu, dass die Erkenntnisse von Head und Mackenzie den Generationen von Student*innen, die diese Darstellungen gelernt haben, unbekannt sind. Das hat mehrere Konsequenzen. Für Studierende bzw. Ärztinnen und Ärzte entsteht der Eindruck, der von Patient*innen beschriebene Spontanschmerz bzw. der im Rahmen der Palpation gewonnene Schmerzbefund wäre eindeutig einem Organ zuzuordnen.

Zwar gibt es klinische Häufigkeiten, sodass bei einem großen Teil der Fälle die Zuordnung zutreffend ist. Jedoch ist die Grundlage dieser Häufigkeiten keine eindeutige oder gar eine eineindeutige Zuordnung aufgrund einer vermeintlichen 1 Segment-1 Organ-Zuordnung, sondern klinische Erfahrung. Patient*innen, bei denen nicht der häufige Fall vorliegt, werden damit gegebenenfalls falsch diagnostiziert und womöglich falsch behandelt, was negative Auswirkungen haben kann.

Zudem trägt die vereinfachte Darstellung übertragener Schmerzen sicherlich zu einer prinzipiellen Verflachung des Verständnisses der Schmerzphysiologie bei. Aus den Arbeiten von Head und Mackenzie lässt sich die Erkenntnis gewinnen, dass alle inneren Organe mit mehreren Spinalnervensegmenten in Verbindung stehen und umgekehrt und dass die den inneren Organen zugeordneten Spinalnervensegmente sich überlappen und eine große Streuung zeigen. In der Konsequenz ist eine Schmerzzuordnung bei Erkrankungen der inneren Organe nie eindeutig.

Im deutschsprachigen Raum sind vor allem die älteren Arbeiten von Hansen und Schliack zu diesem Thema zu erwähnen [8]. Eine vergleichsweise aktuelle Arbeit stammt von Jänig [9].

Die von Head beschriebenen Maximalpunkte verdienen zudem weitere Untersuchung, wie Henke und Beissner in ihrer Arbeit [4] betonen. Hier wäre zu klären, inwieweit deren weitere Untersuchung und die Anwendung der Erkenntnisse zu einer Verbesserung der Methoden der physikalischen Therapie bei der Behandlung akuter und chronischer Schmerzzustände beitragen können.

## Bedeutung von Head für die Neurologie

Während die „Head-Zonen“ fast ein allgemeiner Begriff in der Medizin geworden sind, ist die Bedeutung von Head für die Neurologie heute weniger präsent, aber vielleicht noch wichtiger. Im Folgenden sollen daher die neurologischen Arbeiten von Head chronologisch dargestellt werden. Hier können drei Kernthemen unterschieden werden: Dermatome (1900), Pathophysiologie peripherer Nervenläsionen (1903) und die Neuropsychologie/Aphasiologie (1920).

## Dermatome (1900)

Die Dermatomdarstellungen gehören seit Jahrzehnten zum Kanon der Neuroanatomie und Physiologie. Darüber hinaus sind sie ein klassisches didaktisches und gleichzeitig klinisch nützliches Werkzeug der Neurologie. Head, dessen Schwerpunkt immer die Sensorik war, hat hier zu einem kritischen Zeitpunkt, als die Physiologie der Sensibilität herausgearbeitet wurde, den wahrscheinlich wichtigsten Beitrag geliefert.

Ausgangspunkt bei diesem Thema waren Vorarbeiten von Jean-Martin Charcot (1825–1893) und Silas Weir Mitchell (1829–1914), die sensible Querschnittssyndrome beschrieben [10–13]. Erste Dermatomkarten wurden bereits von M. Allen Starr 1892 und William Thorburn 1893 veröffentlicht [14]. Heads und Campbells Arbeit 1900 war jedoch weitaus breiter angelegt. Sie umfasste 170 Seiten und 392 klinische Fälle [15]. Im Kern handelt es sich um ein Review von 392 pathologisch aufgearbeiteten Herpes-zoster-Manifestationen. Detailliert werden die bekannten Arbeiten zu dem Thema mit Grunderkrankung (meist Malignom, TBC) und der Verteilung der Effloreszenzen (Trigeminusgebiet, zervikal, thorakal und lumbal) beschrieben. Dann folgen 21 Fälle aus der eigenen Klinik. Das Krankheitsbild Zoster bzw. die „Gürtelrose“ war ja schon bekannt [16]. Auch die Histologie war zu Beginn des 20. Jahrhunderts schon gut entwickelt. Bis das dazugehörige Virus identifiziert werden konnte, sollte es aber noch bis 1958 dauern [17].

Head wunderte sich vermutlich sehr darüber, welches Agens wohl spezifisch die Hinterhornganglienzellen befallen könnte. Er beschreibt die klinischen Verläufe, stellt Vergleiche zum viszeralen Schmerz her und stellt Vermutungen zur Pathophysiologie an. Es fällt ihm auf, wie selektiv das Hinterhorn und meist nur genau ein Ganglion betroffen ist. Head schloss eine bakterielle Infektion aus, konnte aber natürlich mit seinen Methoden das Virus noch nicht nachweisen. Er vergleicht das Krankheitsbild mit der ihm bekannten Polio, bei der fast spiegelbildlich das Vorderhorn betroffen ist, aber dann über mehrere Segmente.

**Figure 2:**
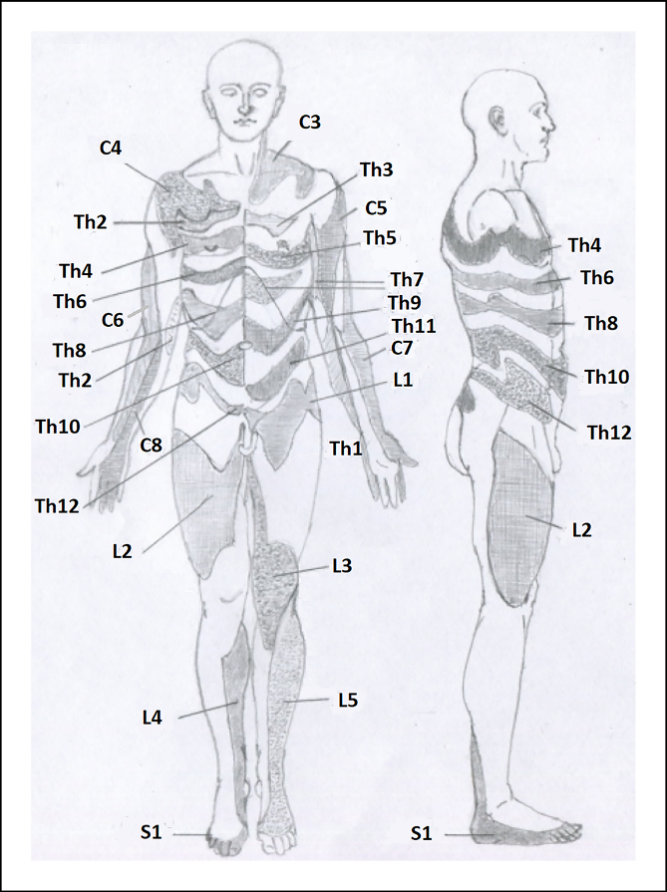

Aus den Fällen konstruierten Head und seine Mitarbeiter in dem Beitrag ein Diagramm, das in wesentlichen Zügen heute als Dermatomkarte bekannt ist (Abb. 2). Die Eigenschaft des Herpesvirus, fast immer nur genau eine sensible Ganglienzelle zu befallen und sich antidrom zur Haut auszubreiten, bewirkt sozusagen eine anatomische eindeutige Markierung. Dies kann umgekehrt klinisch genutzt werden, um die Lokalisation einer sensiblen Läsion auch anderer Ätiologien vorherzusagen. Es ist Heads akribischer Arbeit zu verdanken, den Zusammenhang zwischen Zoster und befallener sensibler Wurzel herzustellen. Da er um das Jahr 1900 einer der einflussreichsten Neurolog*innen und mit der deutschen Nervenheilkunde gut vernetzt war, ist erklärbar, dass sich Heads „Dermatome“ kurz darauf schon überall als das klinische Hilfsmittel durchgesetzt haben, als das wir sie heute kennen.

## Periphere Nervenläsionen (1903)

Ein Meilenstein in der Pathophysiologie peripherer Nervenläsionen ist sicher Heads legendärer Selbstversuch, als er sich 1903 von Henry Percy Dean (1864–1931), James Sherren (1872–1945) und William Halse Rivers (1864–1922) seinen eigenen linken N. radialis (Ramus superficialis) durchtrennen ließ, um differenziert Verlust und Regeneration epikritischer und protopathischer Sinnesqualitäten zu untersuchen [18]. Im Spontanverlauf wurde die Rekonstitution der sensiblen Funktionen über viele Monate detailliert dokumentiert. Diese Arbeiten haben deutlich zum Verständnis des klinischen Verlaufs und der Regenerationsfähigkeit peripherer Nervenläsionen beigetragen.

## Neuropsychologie und Aphasiologie (1920)

Weniger erfolgreich war Head aus heutiger Sicht in dem Bereich, den wir heute Neuropsychologie nennen. Heads Interessen galten der Sensibilität und Neuropathologie. Sicher waren ihm die Aphasietheorien von John Hughlings Jackson bestens bekannt, da dieser sie 1915 in *Brain* ausführlich dargestellt hatte [19]. Bereits kurz nach Beginn des Ersten Weltkriegs untersuchte Head Veteranen mit Verletzungen des Zentralnervensystems. Das Hospital wurde bereits 1919 wieder geschlossen [20], vermutlich weil die Veteranenversorgung danach an anderen Zentren stattfand.

Um die späteren Arbeiten von Head besser zu verstehen, muss man sich vergegenwärtigen, dass um 1920 sowohl Broca (1824–1880) als auch Wernicke (1848–1905) lange schon tot waren. Eine neue Generation neuropsychologisch interessierter deutscher Kliniker wie Brodmann (1868–1918) oder Kleist (1879–1960) entwickelten die klassische Lokalisationstheorie ausgehend von der Aphasie aber weiter. Parallel nahm die Psychometrie bzw. objektive Messung höherer kognitiver Funktionen und die Intelligenz [21] mitsamt der dazugehörigen psychologischen Statistik Fahrt auf.

Die englische Schule von Jackson und Head ging unverändert davon aus, dass nur einfache Funktionen wie Sensibilität und Motorik anatomisch zugeordnet werden können. Der streng lokalisatorischen Sichtweise höherer Funktionen wie Sprache und Gedanken standen sie ablehnend gegenüber. Jackson hat eine eigene psychologische Nomenklatur von Aphasien entwickelt. Diese setzte sich aber nie durch und ist heute deshalb weitgehend unbekannt. Die Sprache als „motorisches Symbol“ der Gedankenwelt war für Jackson und Head nicht lokalisierbar, sondern durch das Gehirn in seiner Gesamtheit repräsentiert.

Was der Neurologe heute als Aphasie betrachtet und der Psychiater vielleicht als Denkstörung, war für Jackson und Head noch ein und dasselbe. Ihre feste Überzeugung war, dass eine „Fakultät“ für Sprache oder Gedächtnis bzw. für höhere Hirnfunktionen wie das Denken schlicht nicht existieren kann [22]. Als konkretes Beispiel führte Jackson an, dass aphasische Patient*innen meist auch nicht schreiben könnten, also einfach ihre Gedanken nicht mehr ausdrücken können.

So erklärt sich der erbitterte Widerstand von Jackson und Head gegen die „Diagramm-Macher“ [23]. Broca sei ein fundamentaler Irrtum unterlaufen. Wenn überhaupt, dann sei eine Aphasie im Corpus striatum zu lokalisieren, schrieb Jackson 1878 in der Erstausgabe von *Brain* [24].

Im Gegensatz zu Jackson, noch ein Neurologe des 19. Jahrhunderts, sprach für Head auch der damalige Zeitgeist. Das frühe 20. Jahrhundert ist aus epistemologischer Sicht auch als dialektische Gegenbewegung zur strengen positivistisch-reduktionistischen Wissenschaft des 19. Jahrhunderts zu sehen [25]. En vogue war nicht mehr die strenge Histologie Virchowscher Schule, sondern mehr aus heutiger Sicht weitschweifige philosophisch-psychologisierende Weltbilder. Während Pearson, Binet und andere Gründerväter der Psychologie schon wissenschaftliche statistische Korrelationsmethoden entwickelten, steckten die Neurolog*innen noch stark in ihrer Methodik endloser Fallbeschreibungen.

Die Psychiatrie begann in diesem Kontext ihre eigenen Methoden zu entwickeln. So ist auch besser erklärbar, dass Jaspers die Lokalisationslehre als „Hirnmythologie“ kritisierte und im psychopathologischen Befund fortan philosophische Aspekte eine größere Rolle spielten [26]. Bei Sigmund Freud hatte die Abwendung von der Hirnforschung und Lokalisationslehre [27, 28] vermutlich mehr pragmatische Gründe, da er als niedergelassener Arzt keine wissenschaftlichen Karrierechancen mehr für sich sah.

Das Problem dieser Gegenbewegung blieb bis heute, dass keine Methodik ihre Hypothesen und Modelle untermauern konnte. EEG und funktionelles MRT, also Methoden, die eine Erfassung der Gehirnaktivität sowie der Morphologie auch an Gesunden erlauben, waren lange noch nicht erfunden. Dennoch erlebten die neuropsychologischen Kritiker von Wernicke und Broca einen Aufschwung. Die „Anti-Lokalisationalisten“, allen voran Marie (1853–1940; [29]), Hughlings Jackson und dann in seiner Nachfolge Head, verhöhnten die „alte“ Schule der klassischen Neurologie, hatten aber aus heutiger Sicht auch keine echte Alternative anzubieten und sind deshalb heute in der Neuropsychologie kaum bekannt.

Der Erste Weltkrieg mit der großen Anzahl von Überlebenden schwerer Hirnverletzungen lieferte für Head und Kollegen neues Anschauungsmaterial für die Untersuchung komplexer Hirnfunktionen, vor allem der Sprache. So ist es auch zu erklären, dass Head aphasische Patient*innen mit einer komplexen, umfassenden und zeitraubenden Batterie selbstentwickelter neuropsychologischer Tests untersucht. Interessanterweise findet sich unter den Verfahren bei Head auch die Erstbeschreibung des Uhrentests [30], womöglich hat er Anleihen bei Binet genommen. Wie zu dieser Zeit üblich, wurden die Tests nie an Gesunden validiert, es wurden keine statistischen Korrelationen durchgeführt, es wurde nicht standardisiert. Die quantitativen Methoden in der Neuropsychologie entwickelten sich dann erst nach dem Zweiten Weltkrieg [31].

Nach seiner Emeritierung und bereits erkrankt, entwarf Head ab 1920 dann als „Alterswerk“ eine eigene Klassifikation von Aphasien. Hier baute er sicher auf den Vorarbeiten von Jackson auf, in dessen Linie er zu sehen ist [32].

Er unterteilte die Sprachstörungen in den Kategorien verbal, syntaktisch, nominal und semantisch. Er selbst sah diese Einteilung aber mehr als innovative neuropsychologische Grundlagenarbeit ohne praktischen Nutzen [33]. Head scheint sich hier – aus heutiger Sicht – überschätzt zu haben mit seinem Anspruch, Ordnung in das unübersichtliche Feld der Aphasiologie zu bringen. Heads monumentale, überlange Werke zum Thema (Band 1: 566 Seiten, Band 2: 466 Seiten) holen auch für damalige Verhältnisse weit aus. Die „Diagramm-Macher“ werden kurz und kritisch behandelt. Die eigenen Aphasiekonzepte werden dagegen hochredundant dargestellt, sind aber teils in sich selbst widersprüchlich. Konzepte wie Alexie, Agraphie und Agraphie lehnte Head ab, ebenso wie die Berücksichtigung der Händigkeit.

Einfluss und Name von Head waren aber ausreichend, um seine Aphasielehre in zeitgenössische neurologische Lehrbücher aufzunehmen [34]. Als spätere, relative Holisten sind noch Kurt Goldstein (1878–1965) und Alexander Lurija (1902–1977) aufzufassen.

Es wäre aber zu kurz gegriffen, Head nur als reinen Anti-Lokalisationalisten zu sehen. So war Head durchaus klar, dass auch seine „Wortzentren“ sich mit der Zuordnung von Wernicke und Broca durchaus überlappten [30]. Für interessierte Leser findet sich eine differenzierte Abwägung des Konflikts bei Norman Geschwind (1926–1984; [35]).

Neben Geschwind, der schließlich Heads Theorien richtigerweise verwarf, bedurfte es noch einer neuen Generation statistisch gut ausgebildeter Neuropsychologen und Verhaltensneurolog*innen, die die heute gängigen Methoden entwickelten. Auch die klinische kognitive Neurologie ist nach Head wieder zu eher einfachen und nützlichen Heuristiken und Ikonen zurückgekehrt, wie sie die Wernicke- oder Broca-Aphasie bedeuten.

Dies änderte sich erst, als die Forschung um organische Psychosyndrome und Demenzen psychometrische Methoden, wie den Mini-Mental-Test, aufnahm. In Deutschland ist hier die Gruppe um Walter Huber und Klaus Poeck zu erwähnen, die sich in den 1970er bis 1980er-Jahren dem Thema Aphasie mit Methoden der modernen Statistik näherte [36].

Vor allem die Diskussion um die Broca-Aphasie währt aber in gewisser Weise bis heute fort [37]. Dies ist kaum verwunderlich angesichts der vor allem neueren Erkenntnisse um Komplexität und Plastizität der an Sprachbildung und Sprachverständnis beteiligten fokalen Hirnregionen sowie nichtlokalisierter neuronaler Netze. Es bleibt zudem abzuwarten, inwieweit der Einsatz künstlicher Intelligenz zur Analyse großer Mengen an funktionellen Bildgebungs- und histologischen Daten diesbezüglich neue Erkenntnisse bringt.

## Fazit

Henry Head ist in der Linie von John Hughlings Jackson zu sehen. Zu Recht war er sein Nachfolger und zu Beginn des 20. Jahrhunderts einer der bedeutendsten Neurolog*innen weltweit. Heute wird sein Name vor allem mit den Head-Zonen in Verbindung gebracht, auch wenn die ersten Darstellungen wohl gar nicht von ihm stammen. Seine eigentlich bahnbrechenden Arbeiten leistete er aber im Bereich der Regeneration nach peripheren Nervenläsionen und in der Charakterisierung der Dermatome. Seine späteren Arbeiten in der Neuropsychologie spiegeln die gegensätzlichen Positionen (lokalisationistisch vs. holistisch) in der Deutung höherer Hirnfunktionen von damals wider. Sie gelten heute als weitgehend überholt und sind nur noch von historischer Bedeutung.
